# The absence of an Atlantic imprint on the multidecadal variability of wintertime European temperature

**DOI:** 10.1038/ncomms10930

**Published:** 2016-03-15

**Authors:** Ayako Yamamoto, Jaime B. Palter

**Affiliations:** 1 Department of Atmospheric and Oceanic Sciences, McGill University, 805 Sherbrooke Street West, Montreal, Quebec, Canada H3A 2K6; 2 Graduate School of Oceanography, University of Rhode Island, Narragansett Bay Campus, Narragansett, Rhode Island 02882, USA

## Abstract

Northern Hemisphere climate responds sensitively to multidecadal variability in North Atlantic sea surface temperature (SST). It is therefore surprising that an imprint of such variability is conspicuously absent in wintertime western European temperature, despite that Europe's climate is strongly influenced by its neighbouring ocean, where multidecadal variability in basin-average SST persists in all seasons. Here we trace the cause of this missing imprint to a dynamic anomaly of the atmospheric circulation that masks its thermodynamic response to SST anomalies. Specifically, differences in the pathways Lagrangian particles take to Europe during anomalous SST winters suppress the expected fluctuations in air–sea heat exchange accumulated along those trajectories. Because decadal variability in North Atlantic-average SST may be driven partly by the Atlantic Meridional Overturning Circulation (AMOC), the atmosphere's dynamical adjustment to this mode of variability may have important implications for the European wintertime temperature response to a projected twenty-first century AMOC decline.

## Abstract (web summary)

The conspicuous absence of a North Atlantic sea surface temperature signature from western European temperature records remains an anomaly. Here, the authors show that this is due to a dynamic atmospheric circulation anomaly that suppresses the expected thermodynamic response.

## Graphical abstract

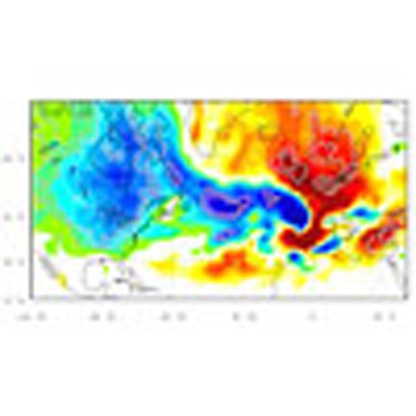

Large-scale, multidecadal variability in North Atlantic sea surface temperature (SST), frequently referred to as the Atlantic Multidecadal Oscillation (AMO), is a prominent feature of Northern Hemisphere climate^1^,^2^: Sahel drought^3^, Atlantic hurricanes^4^, large-scale atmospheric circulation^2^,^5^,^6^,^7^ and summertime European temperature and precipitation^8^,^9^ all respond sensitively to this low-frequency variability in North Atlantic SST. A number of studies suggest that the cause of this SST oscillation is internal variation in ocean heat transport, possibly related to the Atlantic Meridional Overturning Circulation (AMOC) variability^10^,^11^,^12^, with the role of external and/or atmospheric stochastic forcing provoking recent controversy^13^,^14^,^15^,^16^. Evidence in support of the AMO variability being driven internally comes in the form of proxy evidence of a persistent oscillation throughout the past 8,000 years^17^ and the reconstruction of this mode of variability in a number of modelling studies, even in the absence of external forcing^18^,^19^,^20^.

It is well known that the North Atlantic strongly influences western European climate, with the most obvious manifestation being the anomalous wintertime warmth of the region relative to the zonal mean at equivalent latitudes^21^. Moreover, a recent study showed that temporal variability in western European wintertime temperature is set largely by the size of the air–sea turbulent fluxes along the trajectories of Lagrangian air parcels en route to Europe^22^. Coupled with evidence that variability in air–sea heat fluxes over the Atlantic is controlled by the ocean on decadal and longer time scales^23^, it is natural to expect that decadal, basin-scale SST fluctuations should translate to variability in European temperature. Indeed, in all seasons besides winter, the imprint of the AMO is evident in European temperature^8^,^9^. The SST anomaly associated with the AMO persists throughout the year^5^,^10^, making the absence of a wintertime AMO signal in western Europe all the more puzzling (Fig. 1a).

We propose here that the AMO is closely associated with variability in the position and strength of the storm track, which suppresses the influence of the anomalous SST on the heat fluxes seen by Lagrangian parcels transiting to Europe. To evaluate this possibility, we examine the National Centers for Environmental Prediction/National Center for Atmospheric Research 20th-Century Reanalysis (20CR)^24^ from complementary Eulerian and Lagrangian perspectives. The 20CR is one of the longest reanalysis products currently available, and is faithful with independent observations of northeast Atlantic storminess from 1940 onward^25^. Thus, we analyse storm track and Lagrangian pathway variability in the period from 1940 to 2011, which encompasses one full AMO cycle.

## Results

### Eulerian perspective

**Figure 1:**
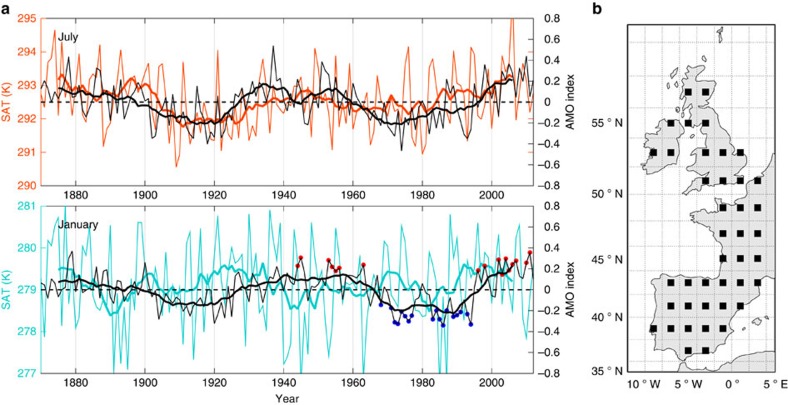
Decadal variability in North Atlantic SST and western European SAT. (**a**) Time series of the linearly detrended North Atlantic SST (black lines, referred to as the AMO index) and SAT averaged over western Europe ([36N 60N] × [10W 3E]; shown in coloured lines) in July (top panel) and January (bottom panel). Bold lines show 10-year running means. The correlation coefficient between the 10-year running mean of the detrended SAT and AMO index is 0.61 in July (statistically significant at 10% confidence level even after accounting for the reduced effective degrees of freedom due to autocorrelation of the time series) and −0.02 in January; these correlations are insensitive to the averaging region chosen for western Europe. The red circles on January plot indicate the AMO-positive years chosen for the composite analysis, whereas the blue circles indicate the AMO-negative years chosen. (**b**) Study region encompassing western Europe ([36N 60N] × [10W 3E]) and locations for the backtracked Lagrangian particle release (black squares).

**Figure 2:**
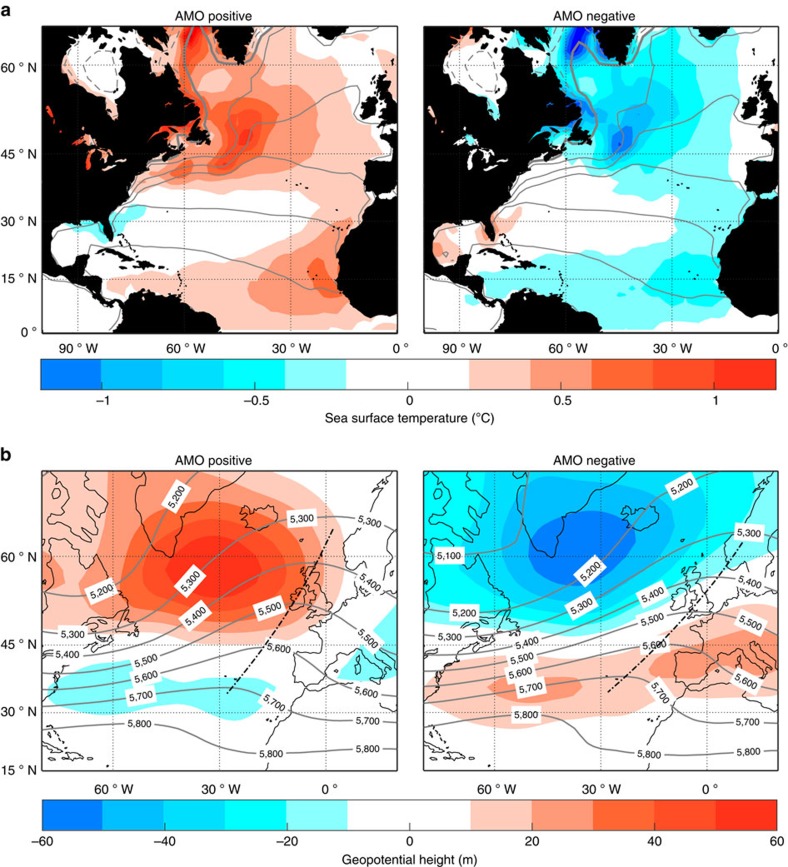
The spatial pattern of the AMO index and its relationship with the atmospheric flow in January. Composite maps of (**a**) sea surface temperature (SST) field and (**b**) 500 hPa geopotential height field (Z500) for AMO anomalously positive years (left panel) and negative years (right panel). The January mean field is shown in contours, and its departure from the 72-year climatology is represented by colour shading. The thick grey contour line in **a** denotes 0 °C, whereas thin (dashed) lines denote positive (negative) SST every 5 °C. The black dashed lines in **b** are drawn through the local maxima of the geopotential height field at each latitude, which is the point where the wind changes direction from south–westerly to north–westerly.

The AMO index, computed by taking an area-weighted mean of the linearly detrended SST field over the North Atlantic^9^,^26^, is generally positive from 1940 to 1963 and 1996 to 2011 and negative from 1966 to 1994 (Fig. 1a). There are various approaches to defining an AMO index^27^,^28^, yet the main features remain almost identical to those identified here regardless of the method chosen^18^,^20^,^27^. In particular, both modelling^6^,^10^,^19^ and observational studies^2^,^6^,^9^,^11^, utilizing different methodologies to isolate only the internal mode of variability, show spatial patterns of SST anomalies similar to that shown in Fig. 2a, and the approximate timing of transitions between AMO phases is not sensitive to its definition^28^.

During each multidecadal period characterized by a given phase of the AMO index, short-term variability gives rise to months in which the basin-averaged SST anomaly is near zero or of the opposite sign relative to the decade in which it is embedded (Fig. 1a). To expose the association of the atmospheric anomaly pattern with SST anomalies, we made composite periods using only the January months with the most extreme AMO index. The extreme AMO months are chosen such that they meet the criteria, |*AMOindex*|>0.15, which is nearly one standard deviation beyond zero (Fig. 1a). All of these extreme months fall within a longer period where the 10-year running mean AMO index has the appropriate sign. In this manner, 17 positive and 18 negative AMO January months are selected (Fig. 1a). We repeated the analysis using all years within the corresponding AMO phase and present the results in the Supplementary Materials, where it is apparent that the key results and interpretation are essentially unchanged, although their statistical power is slightly weaker.

The large-scale atmospheric flow varies with the AMO index (Fig. 2b). The difference in the 500-hPa geopotential height (Z500) field, which is analogous to streamlines, shows that the direction of winds arriving in western Europe changes between the two AMO phases: winds are more northerly during the anomalous AMO-positive years, whereas they are more zonal during the AMO-negative years (Fig. 2b). The more tightly spaced isohypses during the AMO-negative years indicate a swifter flow relative to the AMO-positive years. Accordingly, the AMO-negative years see an elongated and more zonal January storm track (Supplementary Fig. 1), which is consistent with results from a free-running climate model^7^. Composite Z500 maps constructed with more complete sampling of the longer decadal periods associated with the AMO show similar, albeit weaker, anomaly patterns (Supplementary Fig. 2a).

### Lagrangian perspective

**Figure 3:**
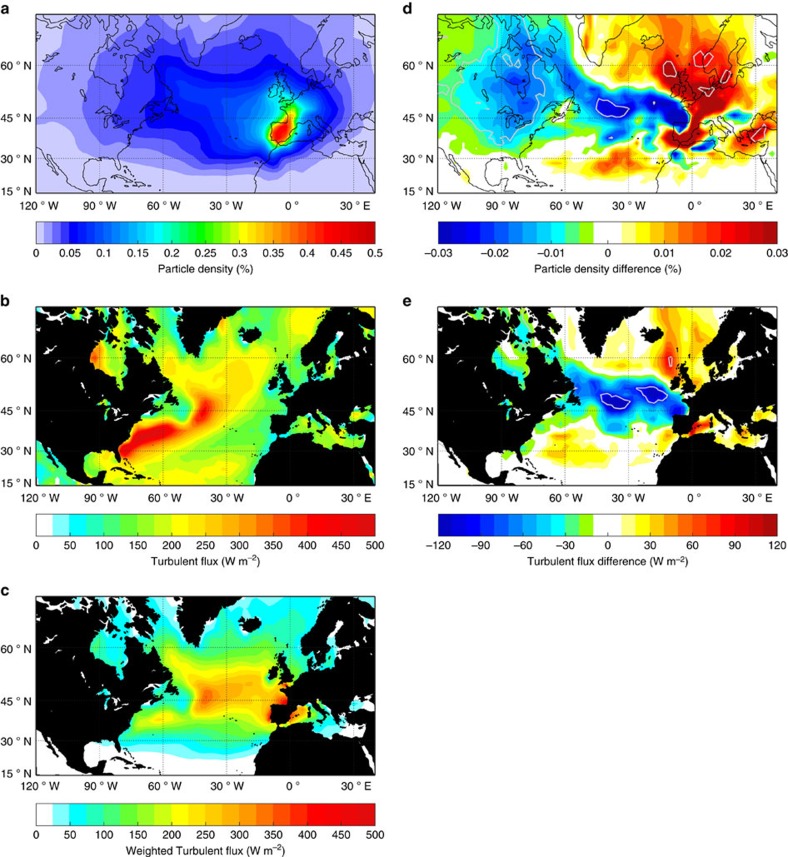
Climatological mean and AMO influence on backward Lagrangian trajectories and the properties along them. (**a**) Lagrangian particle climatological number density, given as the percentage of all hourly positions that were spent in any 2° × 2° grid cell. (**b**) Climatological turbulent fluxes (sensible+latent; W m^−2^) calculated by averaging the fluxes along the Lagrangian trajectories. (**c**) Turbulent fluxes as in **b** but weighted by the fraction of hourly particle positions spent in each grid cell, and normalized to have an equal spatial mean as the unweighted fluxes (see Methods for details; W m^−2^). (**d**) The difference in number density for AMO-positive state minus AMO-negative state (% particle's hourly positions). (**e**) The difference in turbulent fluxes (W m^−2^) for AMO-positive state minus AMO-negative state, weighted and normalized as in **c**. In **d**, statistical significance at 10 and 15% is shown, whereas in **e**, the 15% significance level is shown in grey contours. All the significance levels were obtained using a bootstrapping method (see Methods for details).

The impact of the modulation of the large-scale atmospheric flow on temperature in western Europe is best evaluated in a Lagrangian framework, where the dynamic variability of the atmosphere and the variability in the air–sea turbulent exchange can be assessed simultaneously for the atmospheric particles that influence Europe. Therefore, we launch virtual Lagrangian particles from the surface of forty-one uniformly distributed points over land in western Europe (Fig. 1b) and track them backward in time for 10 days using the atmospheric dispersion model, FLEXPART^29^ (see Methods for details). Ten atmospheric particles are released twice a day in January from 1940 to 2011 from each of the forty-one release points. A climatological two-dimensional histogram of the positions of the resulting Lagrangian particles is shown in Fig. 3a, in which their trajectories are seen spreading out over the North Atlantic, many stretching back to the Labrador Sea and northern Canada. The statistical significance of these results is strengthened when separating the particle launch locations into northern and southern sub-regions of western Europe (see Supplementary Fig. 3 for details).

Our previous work showed that air–sea turbulent fluxes at the base of the atmospheric planetary boundary layer (PBL) govern variability in the potential temperature change along these particle trajectories in January almost entirely: turbulent fluxes alone explain more than 80% of the variability in the potential temperature change along 10-day back trajectories from western Europe^22^. Although the fluxes through the top of the planetary boundary layer are important for closing the heat budget of the layer, they are not crucial for understanding the low-frequency variability of the temperature tendency along Lagrangian trajectories. Therefore, we track the ocean-atmosphere turbulent fluxes along each particle's trajectory by interpolating these fluxes from 20CR to each particle's hourly position when the particle is within PBL. The turbulent fluxes are a function of the temperature and moisture gradients at the air–sea interface and the surface wind speed (see Methods for details). The turbulent fluxes are calculated in 20CR with the product's wind speeds and temperature and moisture gradients, and the size of the fluxes is influenced by correlations between the wind speed and temperatures^22^.

In winter in the North Atlantic, SST is almost always warmer than the surface air temperature (SAT), so the ocean loses heat rapidly to the atmosphere over the entirety of the basin (that is, positive fluxes in our convention; Fig. 3b and Supplementary Fig. 3b). The fluxes over the warm Gulf Stream and its North Atlantic Current extension are generally a factor of five higher than found elsewhere. However, a view of the fluxes weighted by the fraction of time the particles spend in each location on their journey to western Europe (Fig. 3c and Supplementary Fig. 3c) suggests a reduced role of these strong flux regions in establishing western European wintertime temperature.

The difference in the number density of the particle positions between the composite AMO periods (Fig. 3d) shows a significant distinction in the preferred pathways, with the statistical significance increasing when results are separated by particles launched from northern and southern sub-regions of western Europe (Supplementary Fig. 3d). In the AMO-positive years, particles spend more of their 10-day trajectory recirculating locally to the southwest of Iceland. During the AMO-negative years, the pathways are anomalously long, and a greater number of trajectories originate from North America and the Arctic, before transiting over the Labrador Sea and mid-latitude North Atlantic. These differences in the atmospheric trajectories are explained mechanistically by the difference in the Z500 anomalies associated with the AMO, which shows swifter, more zonal winds during AMO-negative years (Fig. 2b). This largely barotropic anomaly pattern has been noted in a number of modelling and observational studies^5^,^10^,^30^,^31^, and is somewhat similar to the atmospheric circulation patterns associated with the North Atlantic Oscillation (NAO)^5^. To explore whether this atmospheric anomaly pattern is linked with anomalous SST conditions of the AMO regardless of the NAO phase, we performed an additional analysis excluding the strong NAO years. This exclusion only amplifies the signal of intensified zonal flow during negative AMO years relative to positive years (c.f. Fig. 3d and Supplementary Fig. 4a). The cause of the linkage between AMO and NAO has been the subject of debate, with several papers arguing that North Atlantic SST anomalies force an atmosperic NAO response^32^,^33^,^34^, and others arguing the reverse^12^,^35^. Regardless of what drives the relationship, the association between the atmospheric circulation and the AMO index is clear in the Lagrangian trajectory composites (Fig. 3d).

The difference map of turbulent fluxes along these Lagrangian trajectories points towards a leading cause of the missing AMO imprint on European wintertime temperatures. During AMO-positive years, the shorter trajectories arriving from the north and southwest (Fig. 3d and Supplementary Fig. 3d) are accompanied by high fluxes (Fig. 3e and Supplementary Fig. 3e). However, the long and zonal trajectories associated with the AMO-negative years are accompanied by even stronger turbulent fluxes over much of the mid-latitude North Atlantic. Therefore, there are partially compensating regions of elevated and depressed flux during both phases of the AMO. The same pattern is found when the difference maps are constructed from composites of the full decadal periods associated with the AMO, but with slightly weaker statistical strength (Supplementary Fig. 2b,c).

**Figure 4:**
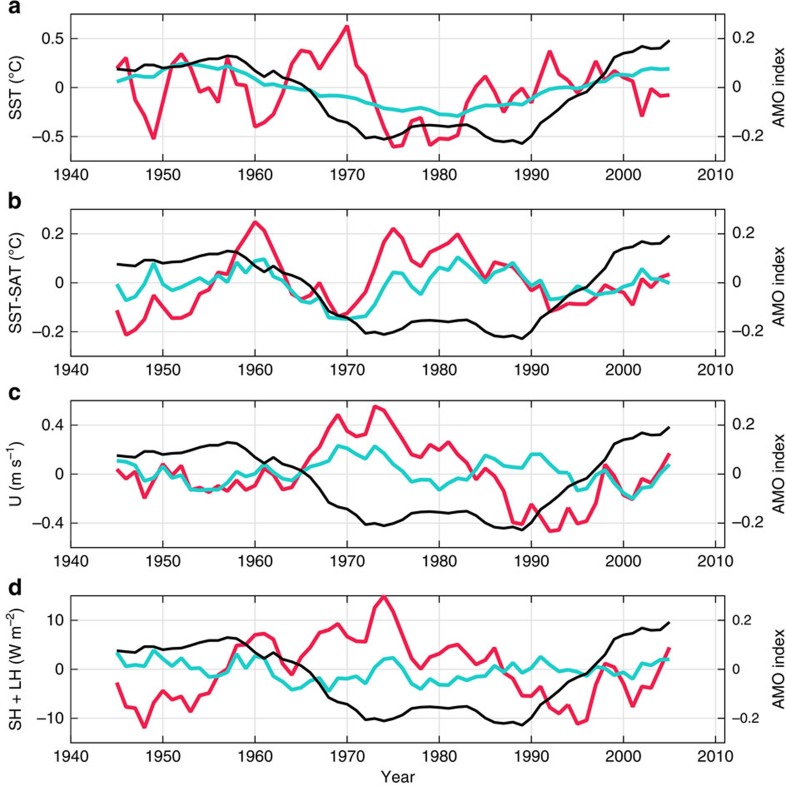
Time series of properties and heat fluxes averaged along Lagrangian trajectories. 10-Year running mean of each linearly detrended variable along true trajectories (red), and along 10 sets of 10 random trajectories (blue), averaged across all 41 release locations. The January 10-year running mean AMO index is overlaid in black in each panel. (**a**) SST (°C), (**b**) SST−SAT (°C), (**c**) wind speed (m s^−1^) and (**d**) turbulent fluxes (W m^−2^).

Time series constructed by averaging along Lagrangian back trajectories (Fig. 4) further reveal the net effect of the combined changes to Lagrangian pathways and the properties along them. Notably, the SST sampled along the Lagrangian trajectories lacks a clear AMO signal (Fig. 4a), because decadal variability in atmospheric trajectories, which travel over a spatially variable SST field, swamps the temporal variability of the North Atlantic average SST. The SAT is highly correlated with the SST (not shown), as turbulent fluxes work to bring the surface boundary layers of the atmosphere and ocean towards equilibrium. Yet, during the negative AMO years from the mid-1970s to 1990, the air advected along Lagrangian trajectories is more anomalously cold than the SST, producing strengthened air–sea temperature gradients (Fig. 4b). Further heightened by stronger winds (Fig. 4c), the largest turbulent fluxes are achieved during these AMO-negative years (Fig. 4d). We acknowledge that at the onset of the AMO-negative period around 1968, both SST and SAT averaged along the trajectories were elevated and fluxes were approximately average. Nevertheless, the overall effect is that the 10-year running mean turbulent fluxes sampled along Lagrangian trajectories are weakly anticorrelated with the AMO index (*r*=−0.39, not significant given the few effective degrees of freedom in the smoothed time series; Fig. 4d). We conclude that, in winter, the dynamic modulation of Lagrangian pathways and the atmospheric properties transported with them oppose the influence of basin-scale SST fluctuations on turbulent air–sea fluxes, thereby concealing the temperature expression of the AMO in atmospheric particles arriving in Europe.

To further assess the degree to which the dynamic modulation of the trajectories is responsible for suppressing the AMO imprint on the fluxes, we re-ran our Lagrangian simulations with randomly selected, unvarying trajectories (see Methods for details). The time series of the 10-year running mean conditions along these random trajectories is plotted in blue in Fig. 4. The SST (Fig. 4a) averaged along these randomly selected trajectories vary in phase with the AMO index, and the anticorrelation of the fluxes with the AMO is eliminated (Fig. 4d). Finally, we confirm that, in summer, when the European temperature reflects AMO variability, the turbulent fluxes vary in phase with the AMO. In July, there is a minimal difference between the preferred pathways by AMO phase (Supplementary Fig. 5). Hence, the dominant signature of the AMO-positive phase appears to be due to higher basin-scale SST, which allows for a broad region of enhanced fluxes, consistent with an extended analysis of air–sea fluxes over the past century^23^.

## Discussion

The strengthening and lengthening of the storm track in sync with anomalously cooler North Atlantic SSTs has important implications for future climate. Given that decadal variability in North Atlantic SSTs may be driven partly by fluctuations in the strength of the AMOC^10^,^11^,^12^, our result suggests the possibility of a stabilizing feedback for ocean circulation: Cooler SSTs associated with a sluggish AMOC is linked with an atmospheric adjustment that enhances turbulent heat fluxes over oceanic convective regions in winter. These larger fluxes could possibly reinvigorate convection, deep water formation and the AMOC. Moreover, the observed link of the atmospheric circulation with the cool SST anomalies of the late 1970s to early 1990s is much like the predicted change of the storm track in response to a decline of the AMOC under global warming^36^. A weakened AMOC has long been thought to cause anomalous cooling in western Europe via a decline in oceanic heat transport and associated atmospheric feedbacks^21^. However, the changes we describe here in atmospheric Lagrangian trajectories and the heat fluxes along them could provide a mechanism that reduces the magnitude of European wintertime cooling on decadal time scales, even as they might stabilize the oceanic circulation.

## Methods

### FLEXPART

We adapted the Lagrangian atmospheric dispersion model FLEXPART version 9.02 (ref. 29) for use with the Twentieth Century Atmospheric Reanalysis product (20CR)^24^ in order to simulate the atmospheric particles released from 41 equally spaced western European locations (Fig. 1b). These release points are chosen from an evenly spaced 2° × 2° grid over the study region [36N 60N] × [10W 3E], when these points fall on land. Every January from 1940 to 2011, ten particles are released from the surface of each of these points twice daily at 0 coordinated universal time (UTC) and 12 UTC and advected backward in time for the duration of 10 days, following the three-dimensional wind field. There are three components to this wind field: (i) resolved wind, (ii) turbulent wind fluctuations and (iii) mesoscale wind fluctuations. FLEXPART accounts for the turbulent wind fluctuations by adding a perturbation to the velocity field for atmospheric particles in the PBL, where these random motions are calculated by solving Langevin equations for Gaussian turbulence. Mesoscale velocity, whose spectral interval falls between the resolved flow and the turbulent flow, is included by solving an independent Langevin equation. The PBL height is diagnosed at each particle's hourly position. The duration of 10 days for the back trajectories was chosen based on the fact that the Lagrangian decorrelation time scale is ∼3 days^22^; thus, the choice of 10 days is long enough for the memory of each particle's initial temperature to be erased under the effect of diabatic processes along the trajectory.

20CR is one of the longest reanalysis products currently available. It has 6-h temporal resolution and 2° × 2° spatial resolution. The product assimilates only observations of surface pressure, monthly SST and sea-ice distributions, and we only use the ensemble mean fields. To assess the reliability of our FLEXPART results with use of 20CR, the trajectories computed using 20CR was compared with those with a default input for FLEXPART, Climate Forecast System Reanalysis (CFSR) forecast and reanalysis data sets from National Centers for Environmental Prediction^37^, which has hourly temporal and 0.5° × 0.5° spatial resolution, for the period of 1981–2009 under the same set up as Yamamoto *et al*.^22^ We found that the trajectory paths computed with 20CR are generally very close to those computed with CFSR especially over the ocean, with particles in 20CR taking slightly more northern paths relative to CFSR (Supplementary Fig. 6). We note that 20CR assimilates monthly mean SST data, whereas CFSR assimilates SST every 6 h. The agreement of the amplitude and variability of the turbulent fluxes along Lagrangian trajectories constructed from the two reanalysis products (Supplementary Fig. 7) suggests that the missing sub-monthly SST variability in 20CR has a minimal impact on these fluxes on interannual and longer time scales.

### Bootstrapping

Bootstrapping was used in order to gauge the statistical significance of the difference in the spatial patterns of Lagrangian trajectory pathways and the fluxes along the trajectories for the two AMO phases (Fig. 3d,e). We sample the Lagrangian particle trajectories or the fluxes along them from randomly selected January months (‘pseudo-periods') for the same number of years as each AMO phase (17 years for AMO positive and 18 years for AMO negative, respectively). We then take a difference between the composite pseudo-periods. This operation was repeated 500 times. We consider differences of the Lagrangian particle density and the fluxes along them from the true AMO composites significant at the 10% (15%) level when this true difference exceeds the 90th (85th) percentile of the pseudo-period differences. We repeated the same procedure to make the composites with entire AMO periods (40 AMO positive years and 29 AMO negative years) and show these results in the Supplementary Materials.

### Surface fluxes along the trajectories

Along the particle trajectories simulated using FLEXPART, the surface turbulent fluxes are interpolated from 20CR sensible heat (SH) and latent heat flux (LH) fields, whenever the particle's hourly position falls within PBL, under the assumption that the turbulent fluxes influence the entire air mass within the PBL. The turbulent fluxes in 20CR are computed using bulk formulae with a typical formulation^38^:


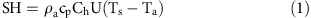



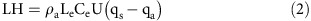


where ρ_a_ is the atmospheric density, c_p_ is the atmospheric heat capacity, C_h_ and C_e_ are transfer coefficients, U is the mean value of wind speed relative to the surface ocean current, T_s_ is sea surface temperature, T_a_ is the atmospheric potential temperature at a reference height, L_e_ is latent heat of evaporation, q_s_ is interfacial value of water vapour mixing ratio, and q_a_ is the atmospheric water vapour mixing ratio at a reference height.

Time series of the mean accumulated surface heat fluxes along the trajectories using 20CR are highly correlated with those using CFSR (Supplementary Fig. 7), with the average correlation coefficient being *r*=0.92.

### Weighting of composite fluxes

In the composite figures of weighted surface fluxes along the trajectories (Fig. 3c,e), weights are proportional to the fraction of all particle positions that pass over a each 2° × 2° grid cell (that is, the number density, given as a percentage in Fig. 3a). These weights are scaled so that the mean value of the climatological map (Fig. 3c) is equal to the mean of the unweighted climatology (Fig. 3b). The spatial mean of both mapped fields used in this scaling includes only those grid cells visited by at least 0.01% of the climatological particle positions; these collectively contain 90.1% of all hourly particle positions.

### Randomly selected trajectories

The unvarying particle trajectory pathways used to produce Fig. 4 (blue lines) were chosen by randomly selecting ten particles from the set of all possible pathways generated from the full 72-year Lagrangian simulation. The truly varying surface fluxes are interpolated along these random trajectories. We then repeat this process ten times, each time by picking a different random set of ten trajectories from each release point, thereby creating a spread of particle positions. In total, 100 particles (10 particles × 10 realizations) are selected for each release location.

## Additional information

**How to cite this article:** Yamamoto, A. *et al*. The absence of an Atlantic imprint on the multidecadal variability of wintertime European temperature. *Nat. Commun.* 7:10930 doi: 10.1038/ncomms10930 (2016).

## Supplementary Material

Supplementary Information Supplementary Figures 1-7 and Supplementary References.
