# Supplementary material for: The absence of an Atlantic imprint on the multidecadal variability of wintertime European temperature
Source: Nat Commun. 2016 Mar 15;7:10930. doi: 10.1038/ncomms10930 (PMC4796317; doi:10.1038/ncomms10930)
Supplement: Supplementary Information — Supplementary Figures 1-7 and Supplementary References. [file ncomms10930-s1.pdf]

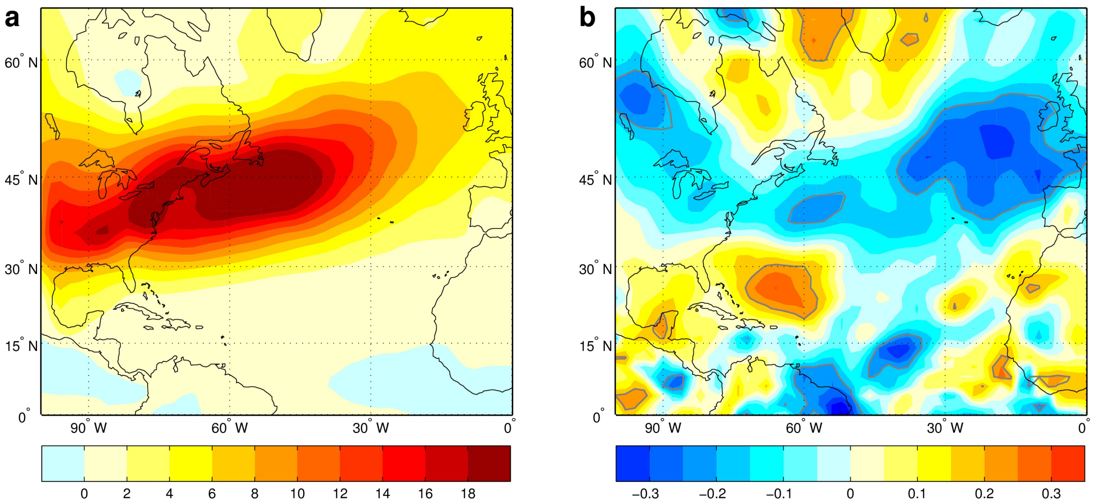

Supplementary Figure 1: **Climatological storm track and storm track modulation associated with the AMO index.** a) Climatological January meridional eddy heat flux ($\overline{v'T'}$), an indicator of the position of the storm track [K m s^{-1}]. The meridional heat flux is computed after band-pass ([2 7] days) filtering the 20CR data for January 1940 - 2011. b) Correlation coefficient between the AMO index and the eddy heat flux shown in a). The regions where $p < 0.1$ are encircled with grey contours. This correlation map is consistent with the results from a free-running climate model, in which warm North Atlantic SST anomalies lead to reduced northward atmospheric eddy heat transport at mid-latitudes[1], although the band of anticorrelation is stronger and more zonal in 20CR than the free-running climate model. The zonal band of negative correlation along 45°N is centered approximately in the core of the storm track and suggests a weakening of the storm track in that region during AMO positive periods. The negative correlation just west of the British Isles and France indicates an eastward elongation of the storm track during AMO negative periods.

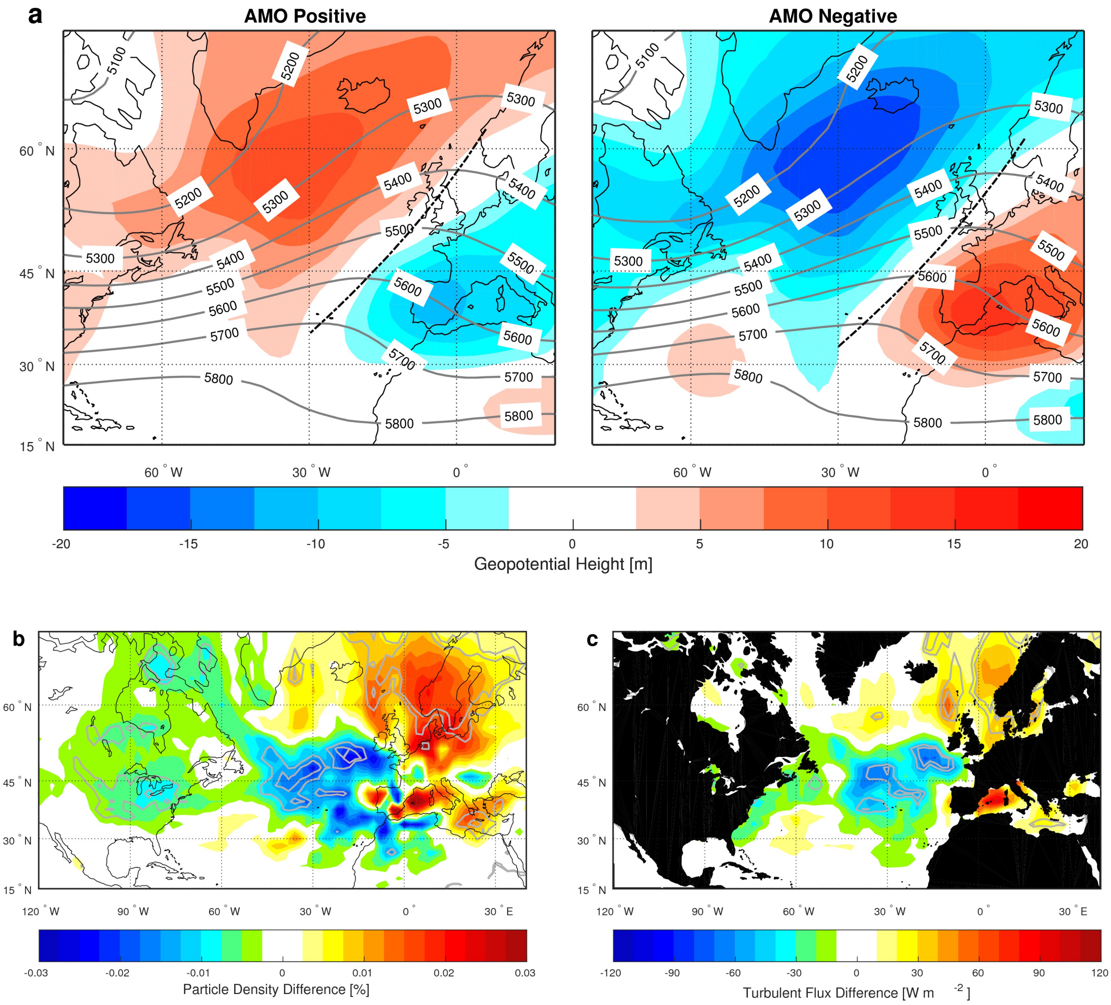

Supplementary Figure 2: **Reproduction of Figure 2b, Figure 3d and 3e in main text, using all continuous years associated with the multidecadal swings of the AMO.** a) 500 hPa geopotential height field (Z500) for AMO positive state (left panel) and negative state (right panel). The mean field is shown in contours, and its departure from the 72-year climatology is represented by colour shading. The black dashed lines are drawn through the local maxima of the geopotential height field along at each latitude, which is the point where the wind changes direction from south-westerly to north-westerly. Note that the colour scale in this panel is different from Figure 2b in the main text. b) The difference in number density for AMO positive state minus AMO negative state [% particle's hourly positions]. c) The difference in turbulent fluxes [W m^{-2}] for AMO positive state minus AMO negative state, weighted and normalised. For panel b) and c), the grey contours indicate 10 and 15% significance level from bootstrapping. The years used to make these composites include 1940 - 1963 and 1996 - 2011 for the AMO positive phase, and 1966 - 1994 for the AMO negative phase.

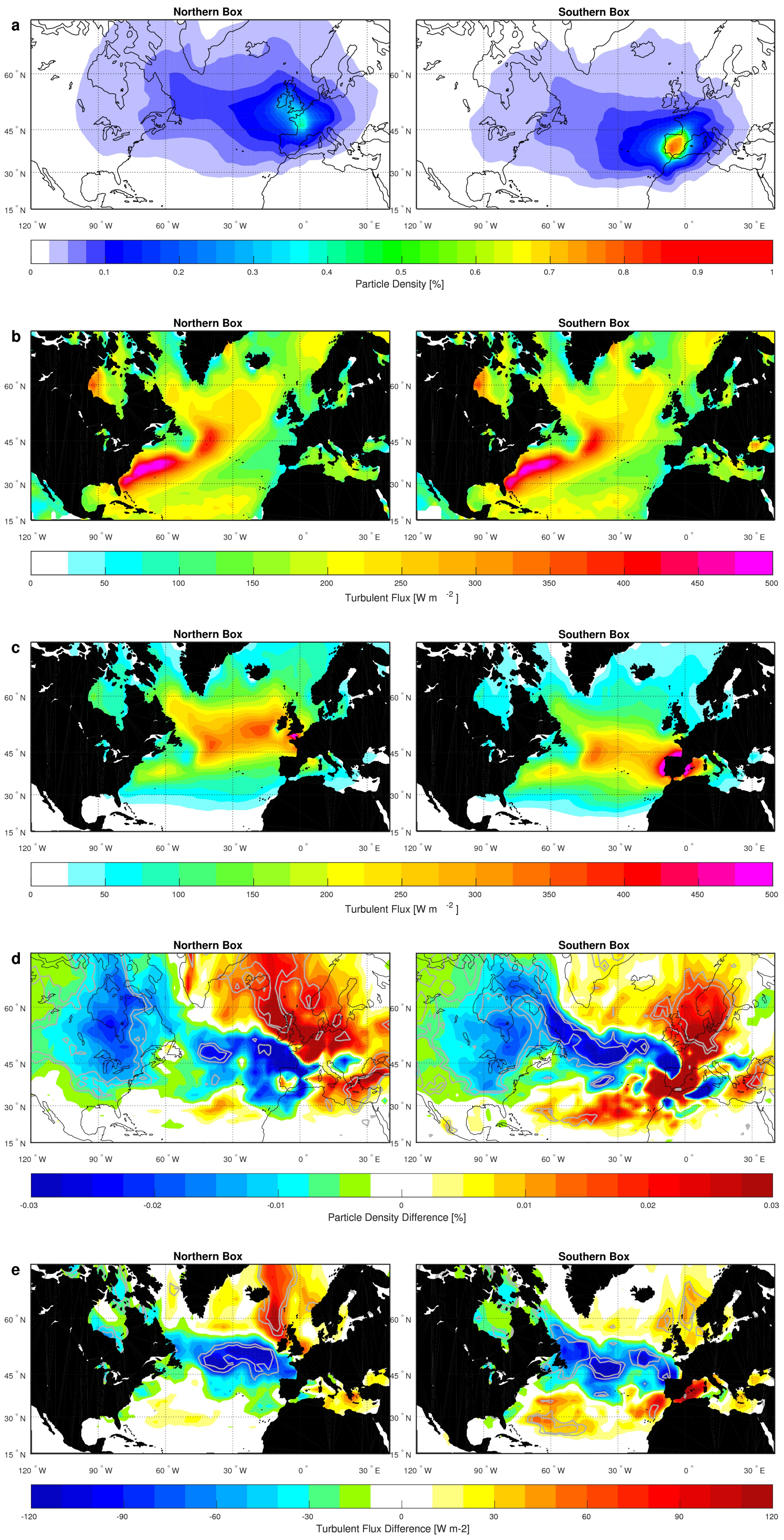

Supplementary Figure 3: **Reproduction of Figure 3 in main text, separately plotted for the northern and southern boxes.** The results from the northern box ([46N 60N]×[10W 3E]) are shown on the left hand panels and those from the southern boxes ([36N 46N]×[10W 3E]) are shown on the right hand panels. a) Lagrangian particle climatological number density, given as the percentage of all hourly positions that were spent in any $2° \times 2°$ grid cell. b) Climatological turbulent fluxes (sensible + latent) [W m^{-2}] calculated by averaging the fluxes along the Lagrangian trajectories. c) Turbulent fluxes as in panel b), but weighted by the fraction of hourly particle positions spent in each grid cell, and normalized to have an equal spatial mean as the unweighted fluxes (see Methods) [W m^{-2}]. d) The difference in number density for AMO positive state minus AMO negative state [% particle's hourly positions]. e) The difference in turbulent fluxes [W m^{-2}] for AMO positive state minus AMO negative state, weighted and normalized as in panel c). In panel d) and e), statistical significance at 10 and 15% is shown in grey contours, which was obtained using a bootstrapping method (see Methods). See Figure 1b in the main text for all launch locations of the backtracked trajectories.

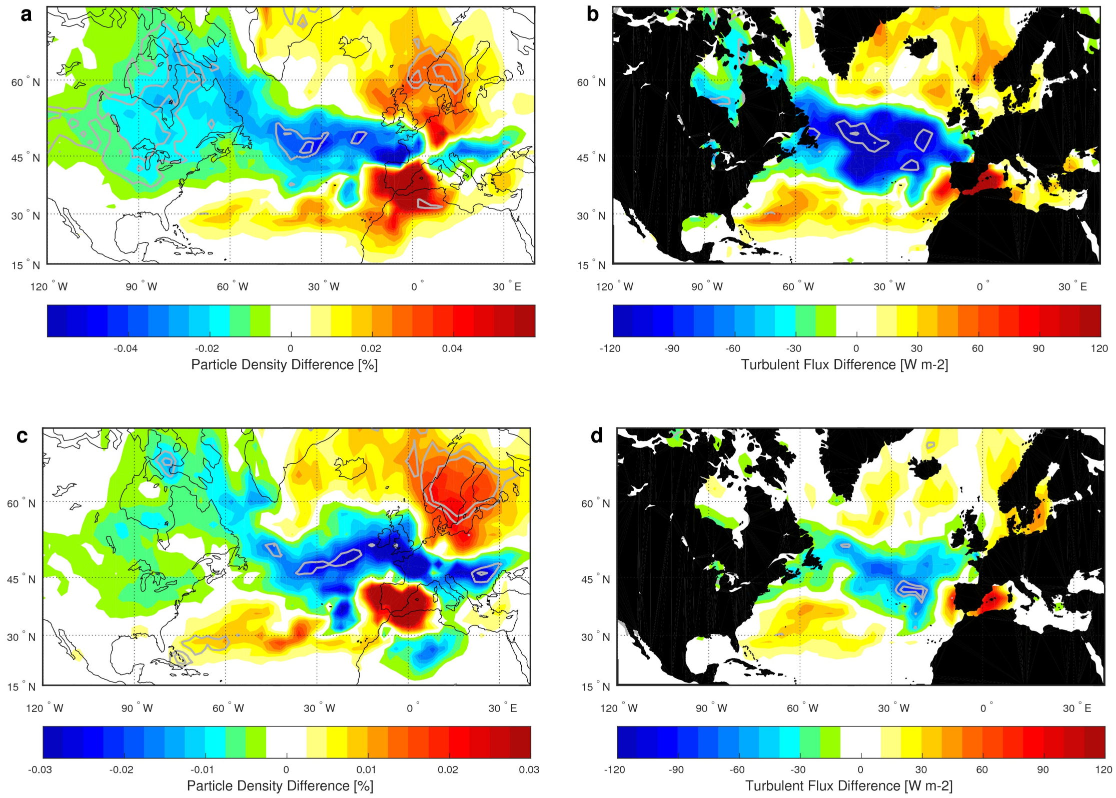

Supplementary Figure 4: **Reproduction of Figure 3d and 3e in main text, excluding NAO extreme years.** a) The mean difference in number density for AMO positive state minus AMO negative state [% particle's hourly positions], made by removing those years from the composites in which the absolute value of the mean January NAO index is greater than 1.8, which is approximately one standard deviation of NAO. In this manner, 11 AMO positive years, and 8 AMO negative years contribute to these composites. Note that the scale range is double of that of Figure 3e in the main text. b) The difference in turbulent fluxes [W m^{-2}] for AMO positive state minus negative state, where the composites exclude the extreme NAO years. c) Same as panel a), but where the AMO composite includes the entire multidecadal periods associated with the AMO (positive from 1940 - 1963 and 1996 - 2011, and negative years from 1966 - 1994), excluding extreme NAO Januaries ($|NAOindex| > 1.8$). Here, 29 AMO positive years and 15 AMO negative years contribute to these composites. d) Same as panel b), but using the same years as panel c). The grey lines indicate 10 and 15% significance level from bootstrapping, but 77 out of 500 of the pseudo-period differences were excluded because their mean NAO index difference was greater than one standard deviation of the sample population.

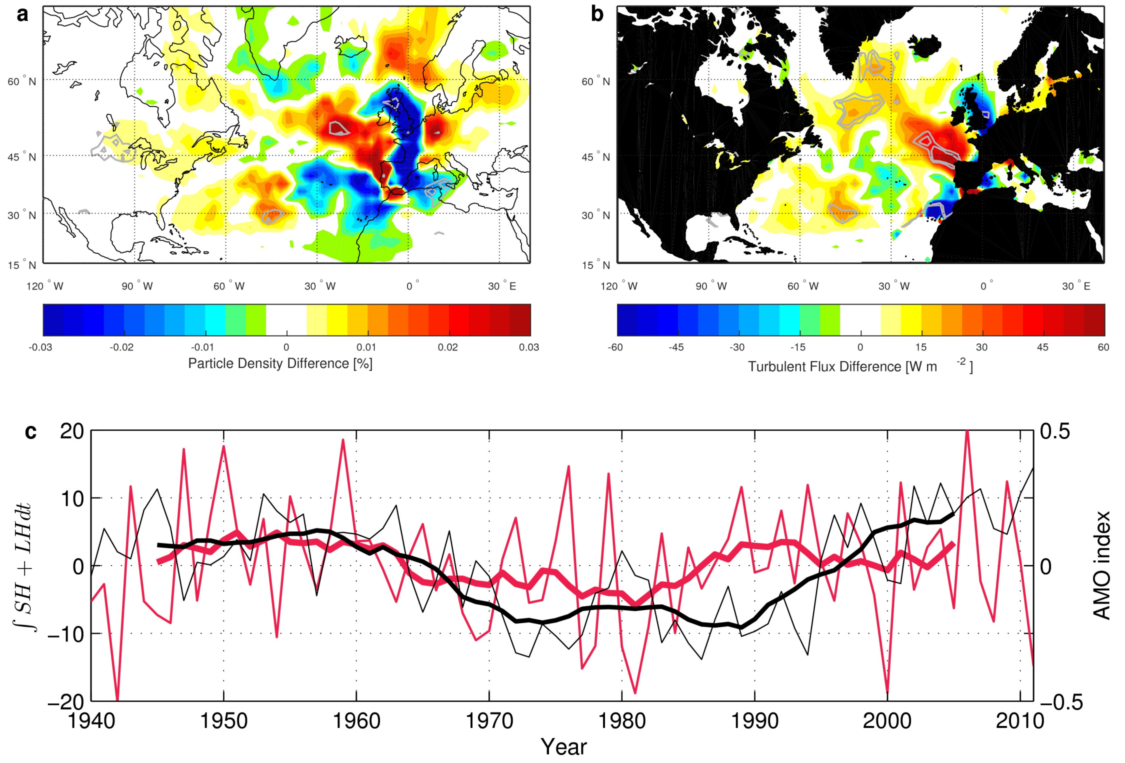

Supplementary Figure 5: **The AMO influence on Lagrangian trajectories to Europe and the air-sea heat fluxes along them in July.** (a) The mean difference in number density for AMO positive state minus AMO negative state [% particle's hourly positions]. (b) The mean difference in turbulent fluxes for AMO positive state minus AMO negative state [W m^{-2}] (c) Time series of the mean detrended 10-day accumulated surface heat fluxes [K] (red coloured lines; thin line for each July, and bold line for 10-year running mean) and AMO index (black lines; thin line for each July and bold line for 10-year running mean). The correlation coefficient between the low-pass filtered surface flux and AMO index is 0.57 ($p = 0.10$, after accounting for the reduced effective degrees of freedom due to autocorrelation of the time series). The grey lines indicate 10 and 15% significance level from bootstrapping.

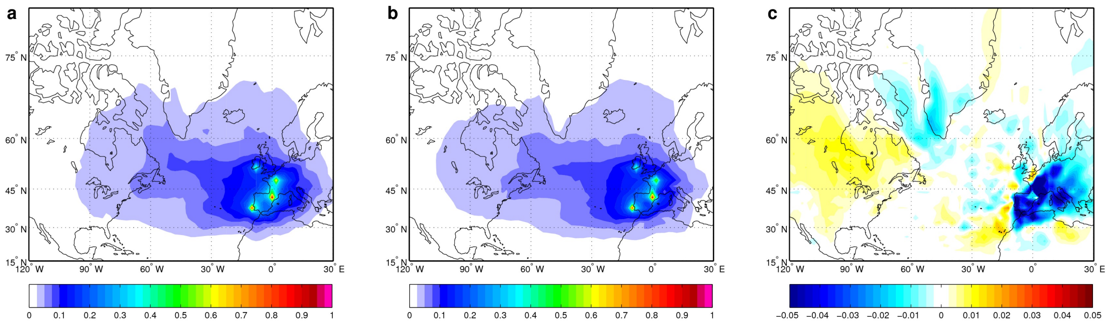

Supplementary Figure 6: **A comparison of Lagrangian trajectories constructed using two different atmospheric reanalysis products.** a) Number density plot of trajectories computed with 20CR for 1981 - 2009 [% particle's hourly positions]. b) Same as a), but for trajectories computed using NCEP Climate Forecast System Reanalysis (CFSR). c) Number density computed with CFSR minus that with 20CR. CFSR is a default input of FLEXPART, and is one of the state-of-art reanalysis products, which assimilates all available conventional and satellite observations, whereas 20CR assimilates only surface pressure, SST, and sea-ice distributions. All the data are constructed using the same set up as Yamamoto et al.[2], in which 50 particles are launched twice a day during January from 4 western European cities.

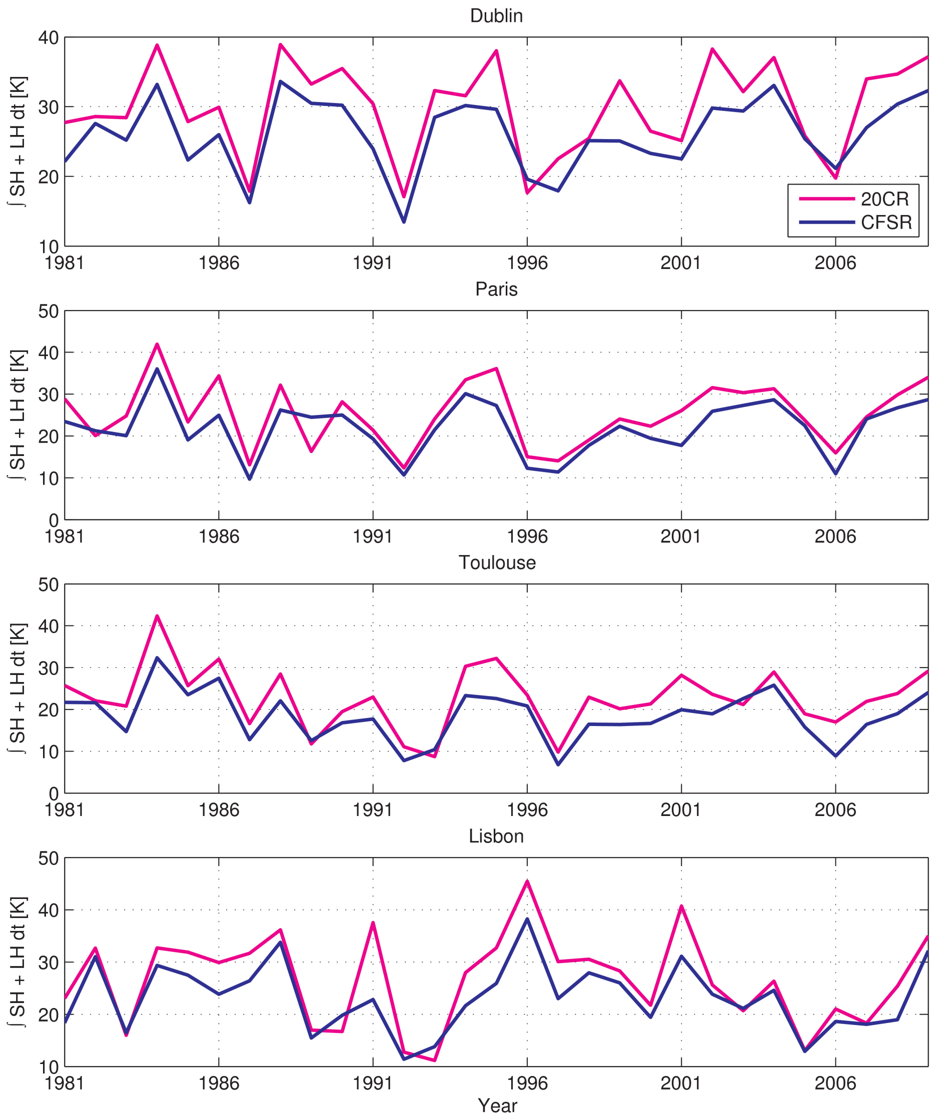

Supplementary Figure 7: **Comparison of the time series of accumulated turbulent fluxes along the trajectories computed with the CFSR and 20CR.** The result using CFSR is shown in blue, and the result using 20CR is shown in magenta. The experiments are done under the same set up as Yamamoto et al.[2]. The turbulent heat fluxes computed with 20CR tends to be higher than that with CFSR by 3.7 K on average, but the interannual variability is almost identical, with the mean correlation coefficient between the two equal to 0.92 (statistically significant at 5% confidence level).

References

[1] Zhang, R. & Delworth, T. L. Impact of the Atlantic Multidecadal Oscillation on North Pacific climate variability. *Geophysical Research Letters* **34**, 2–7 (2007).

[2] Yamamoto, A., Palter, J. B., Lozier, M. S., Bourqui, M. S. & Leadbetter, S. J. Ocean versus atmosphere control on western European wintertime temperature variability. *Climate Dynamics* **45**, 3593–3607 (2015).
